# The Association Between Mandibular Third Molar Impaction, Distal Caries of Second Molars, and Pericoronal Follicle Enlargement: A Retrospective Panoramic Radiographic Study

**DOI:** 10.3390/tomography12090122

**Published:** 2026-08-25

**Authors:** Emine Tuna Demir, Mustafa Cenk Durmuşlar, Aybike Şeker Yılmaz

**Affiliations:** 1 Department of Oral and Maxillofacial Surgery, Faculty of Dentistry, Istanbul Kent University, Istanbul 34433, Turkey; dt.aybikeseker@gmail.com; 2 Department of Oral and Maxillofacial Surgery, Faculty of Dentistry, Biruni University, Istanbul 34015, Turkey; mdurmuslar@biruni.edu.tr

**Keywords:** impacted third molar, follicle enlargement, distal caries, second molar, panoramic radiograph, Winter classification, Pell–Gregory classification

## Abstract

**Background/Objectives**: Impacted mandibular third molars are associated with distal caries of the adjacent second molar and pericoronal follicle enlargement. This study evaluated the independent associations of both Winter angulation and Pell–Gregory classification with these complications on panoramic radiographs. **Methods**: This retrospective radiographic study evaluated 1491 impacted mandibular third molars from 1487 patients aged 18 years or older, reported in accordance with the STROBE guidelines for observational studies. Impaction patterns were classified using both the Winter and Pell–Gregory systems. Follicular space exceeding 2.5 mm was defined as follicle enlargement, measured digitally on panoramic images. Distal caries was defined as a radiolucent lesion on the distal surface of the adjacent second molar. Binary logistic regression (univariate and multiple models) was used to assess associations. **Results**: Among the 1487 patients (745 females, 742 males; mean age 25.21 ± 4.15 years), neither Winter angulation nor Pell–Gregory classification was associated with distal caries in univariate or multivariable analysis (all *p* > 0.05). Pericoronal follicle enlargement was significantly associated with deeper impaction: Pell–Gregory Class I carried lower risk than Class III (OR 0.296; 95% CI 0.184–0.478; *p* < 0.001), and Class A carried lower risk than Class C (OR 0.254; 95% CI 0.161–0.400; *p* < 0.001). Younger age independently predicted follicle enlargement (OR 0.964/year; *p* = 0.028). Winter angulation was not independently associated with follicle enlargement (all *p* > 0.05). **Conclusions**: Neither Winter nor Pell–Gregory classification predicted distal second molar caries on panoramic radiography. Deeper Pell–Gregory impaction positions and younger age were independently associated with pericoronal follicle enlargement, supporting early radiographic surveillance in young patients with deep impactions.

## Simple Summary

Wisdom teeth that stay stuck in the jaw can damage the neighboring tooth and cause a fluid-filled sac to form around them. This study examined X-rays from a large group of adults to see whether the angle or position of a stuck wisdom tooth predicts these problems. Angle did not predict damage to the neighboring tooth, but teeth positioned deeper in the jaw, especially in younger patients, more often developed the fluid-filled sac. These findings suggest dentists should watch X-rays of deeply impacted wisdom teeth in young patients closely, since early detection may help prevent more extensive treatment later.

## 1. Introduction

Impacted teeth are defined as teeth that fail to achieve a functional position in the dental arch despite the completion of root formation, remaining embedded in bone or soft tissue [1]. Mandibular third molars represent the most commonly impacted teeth in clinical practice. A recent systematic review and meta-analysis of 183,828 subjects reported a pooled global prevalence of 36.9% (95% CI: 33.1–40.7%) per subject [2].

The etiology of third molar impaction is multifactorial, including arch length discrepancy, ectopic positioning, obstruction of the eruption pathway, endocrine disorders, and genetic syndromes such as Down syndrome and cleidocranial dysostosis [1,3]. Impacted mandibular third molars are associated with a range of pathological complications, including distal caries of the adjacent second molar, periodontal pocketing, pericoronitis, root resorption, and pericoronal cyst formation [4,5]. The clinical significance of these complications is further underscored by the fact that unaddressed follicle enlargement may progress to dentigerous cyst formation, or in larger lesions, contribute to mandibular angle fractures [6], and that inadequately treated pericoronitis may rarely lead to life-threatening infections such as Lemierre syndrome [7].

The two most widely adopted classification systems are the Winter classification, which categorizes impaction angulation as vertical, mesioangular, distoangular, or horizontal [8], and the Pell and Gregory classification, which describes the available retromolar space (Class I, II, or III) and depth of impaction relative to the adjacent second molar (Class A, B, or C) [9,10,11,12]. Mesioangular impaction accounts for approximately 41% of cases in meta-analytic data [2].

Distal caries of the mandibular second molar is the most frequently reported complication, with a pooled prevalence of approximately 29.89% (95% CI: 21.05–38.74%) in systematic review data [13]. Recent retrospective and multicenter studies have confirmed that mesioangular and horizontal impactions carry the greatest risk, and that detection rates vary according to third molar management strategy and imaging protocol [14,15,16]. Pericoronal follicle enlargement exceeding 2.5–3 mm warrants further evaluation for possible cystic transformation [17]. Dentigerous cysts are reported in approximately 2.1% of impacted third molar extractions and are most frequently associated with deeper impactions [18,19].

Panoramic radiography remains the most widely used modality for the pre-operative assessment of impacted third molars, despite well-documented limitations in sensitivity for proximal caries detection and three-dimensional follicular assessment compared with bitewing radiography or cone-beam computed tomography (CBCT) [20]. Recent CBCT-based investigations have shown that three-dimensional imaging can refine the assessment of both impaction-related bone change and follicular pathology [15,21,22]; while panoramic radiography remains the pragmatic first-line modality for population-level screening, CBCT should be considered as an adjunct whenever a panoramic finding requires confirmation before a management decision is made.

Although individual classification systems have been studied in relation to these complications, few studies have simultaneously evaluated both the Winter and Pell–Gregory systems and both distal caries and follicle enlargement as co-primary outcomes in the same large cohort using multivariable logistic regression. The aim of this study was therefore to evaluate the independent associations of both classification systems with distal second molar caries and pericoronal follicle enlargement, stratified by patient age and sex.

## 2. Materials and Methods

### 2.1. Study Design and Ethical Approval

**Figure 1:**
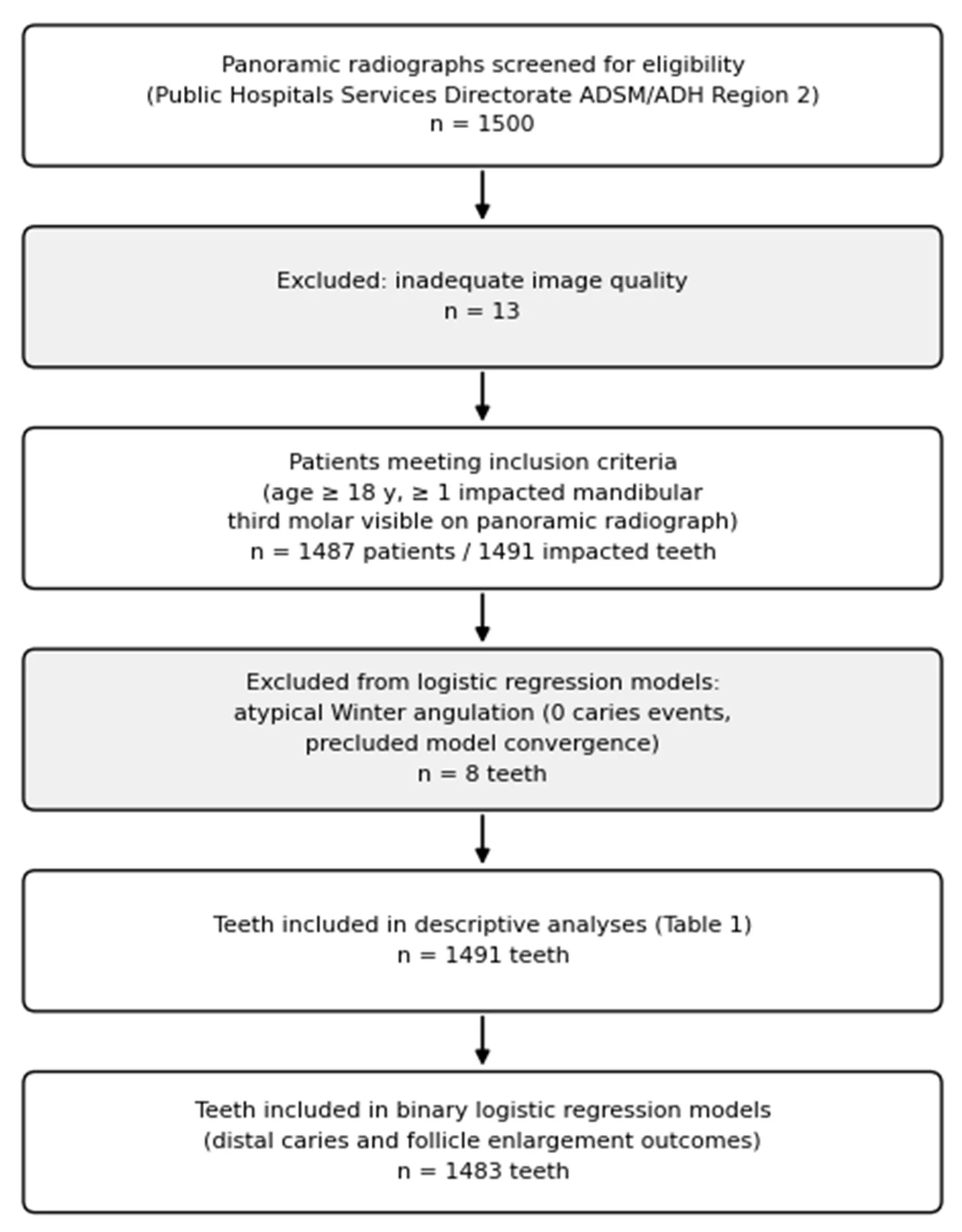
Patient screening and analytic-inclusion flow diagram (STROBE-style).

This retrospective radiographic study was reported in accordance with the Strengthening the Reporting of Observational Studies in Epidemiology (STROBE) guidelines; a completed STROBE checklist is provided in the Supplementary Materials (Table S1), and a flow diagram summarizing patient screening and analytic inclusion is provided in Figure 1. The study was approved by the Biruni University Non-Interventional Clinical Research Ethics Committee (decision no. 2023/77-22, approved 6 January 2023) and was conducted in accordance with the Declaration of Helsinki. Patient consent was waived by the ethics committee owing to the retrospective nature of the study and the use of anonymized radiographic records.

### 2.2. Inclusion and Exclusion Criteria

Inclusion criteria were patients aged 18 years or older with at least one impacted mandibular third molar visible on panoramic radiography, drawn from patients attending the Public Hospitals Services Directorate ADSM/ADH Region 2. Exclusion criteria were fully erupted third molars, restored or crowned second molars, congenital conditions associated with dental anomalies (e.g., cleidocranial dysostosis, Down syndrome), and radiographs of inadequate image quality. The numerical outcomes of the screening and exclusion process are reported in Section 3.1 (Results) and summarized in Figure 1.

### 2.3. Radiographic Assessment

All radiographs were independently evaluated by three examiners from the Department of Oral and Maxillofacial Surgery: two oral and maxillofacial surgeons (E.T.D., Assistant Professor; M.C.D., Professor) and one doctoral candidate in oral and maxillofacial surgery (A.Ş.Y.). Disagreements were resolved through structured consensus discussion. Individual assessor scores were not separately recorded prior to consensus; therefore, formal intra-observer or inter-observer reliability statistics (e.g., intraclass correlation coefficient or Cohen’s kappa) could not be calculated, and the individual contributions of the three examiners could not be compared to characterize systematic differences between assessors. Both limitations are acknowledged in Section 4.4 (Limitations).

Impaction angulation was classified using the Winter classification (vertical, mesioangular, distoangular, or horizontal) [8]. Eight teeth showing atypical angulation were excluded from logistic regression analyses owing to the absence of caries events in this subgroup, which precluded model convergence, but are included in descriptive analyses (Table 1). Depth of impaction was assessed using the Pell and Gregory classification: Class A (occlusal surface at or above the occlusal plane of the second molar), Class B (between the occlusal plane and cervical line), and Class C (below the cervical line). Available retromolar space was classified as Class I (sufficient space), Class II (insufficient space), or Class III (no available eruption space) [9].

Distal caries was defined as a visible radiolucent lesion on the distal surface of the mandibular second molar on panoramic radiography. Pericoronal follicle enlargement was defined as a follicular radiolucency exceeding 2.5 mm in its widest dimension, measured digitally using the integrated measurement tool of the panoramic imaging software, as the maximum distance between the crown surface and the inner cortical border of the follicle. This 2.5 mm threshold follows radiographic criteria widely cited in the panoramic literature for pathological follicular enlargement [17]; we note, however, that the histological validation of this threshold reported by Li et al. [17] was performed on cone-beam computed tomography (CBCT), not panoramic radiography, and that panoramic imaging is subject to variable magnification, projection-geometry distortion, and anatomical overlap. No distortion or magnification calibration and no formal cross-validation against CBCT were performed for the present cohort. Accordingly, the 2.5 mm cutoff used here should be regarded as a pragmatic, literature-based screening threshold for two-dimensional imaging rather than a metrically validated measurement, and this constraint is discussed further in Section 4.4 (Limitations).

### 2.4. Unit of Analysis and Statistical Considerations

Analyses were conducted at the tooth level, as impaction characteristics and outcomes were recorded per tooth. Of the 1487 patients, 4 contributed bilateral impacted third molars (0.27%); the remaining 1483 contributed a single tooth. This bilateral-impaction rate is lower than typically reported in hospital-based adult populations, which may reflect the inclusion criterion requiring only one radiographically visible impacted tooth per patient, prior unilateral extraction not captured in the retrospective record, or local referral patterns at the source institution; this possible selection effect is acknowledged in Section 4.4 (Limitations). While teeth from the same patient are not fully independent, the minimal bilateral contribution limits the practical impact of clustering; this is nonetheless acknowledged as a methodological limitation, and future studies should employ multilevel modeling or generalized estimating equations.

### 2.5. Statistical Analysis

Data were analyzed using IBM SPSS Statistics Version 23 (IBM Corp., Armonk, NY, USA). Binary logistic regression analysis, reported as univariate and multiple (adjusted) models, was used to assess associations between impaction pattern, patient sex, age, and the two outcomes. Results are presented as mean ± standard deviation (SD) for continuous variables and as frequencies and percentages for categorical variables. Odds ratios (ORs) and 95% confidence intervals (CIs) are reported. Statistical significance was set at *p* < 0.05. Borderline findings (0.05 ≤ *p* ≤ 0.10) are discussed where clinically relevant.

## 3. Results

**Figure 2:**
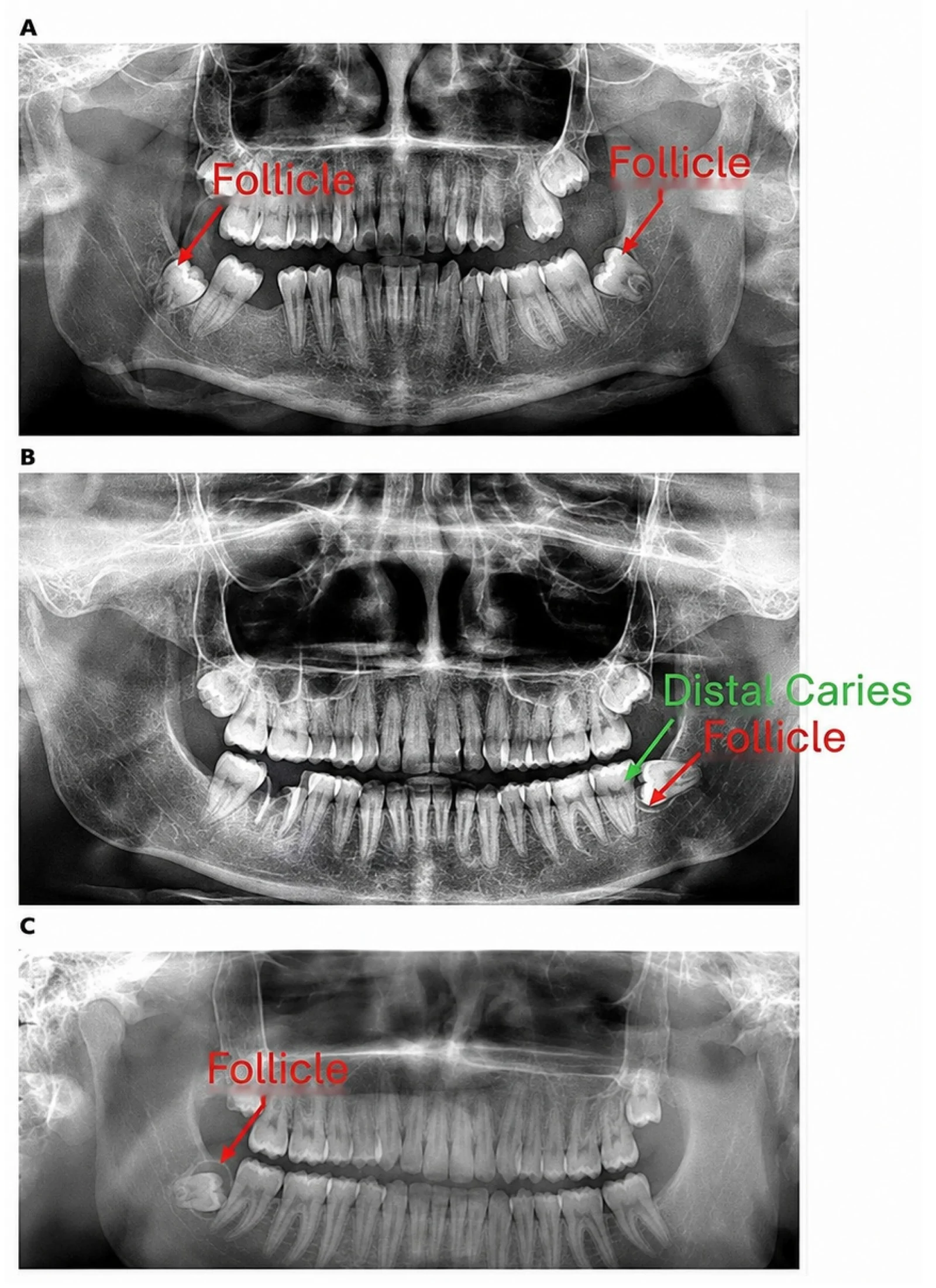
Representative panoramic radiographs illustrating impaction patterns and associated findings. (**A**) Bilateral mandibular third molar impaction with mesioangular angulation on the right (tooth 38) and horizontal impaction on the left (tooth 48), with pericoronal follicle enlargement indicated bilaterally (arrows). (**B**) Distoangular impacted mandibular third molar (tooth 38) with a radiolucent lesion on the distal surface of the adjacent second molar (tooth 37) consistent with distal caries (green arrow), and associated pericoronal follicle enlargement around tooth 38 (red arrow). (**C**) Deep mandibular third molar impaction (Pell–Gregory Class C) with enlarged pericoronal follicular space (tooth 48; arrow).

Representative examples of the radiographic findings evaluated in this study—bilateral follicle enlargement associated with mesioangular and horizontal impaction, a distoangular impaction with adjacent distal caries, and a deep (Pell–Gregory Class C) impaction with enlarged follicular space—are shown in Figure 2.

### 3.1. Study Sample

A total of 1500 panoramic radiographs from patients attending the Public Hospitals Services Directorate ADSM/ADH Region 2 were initially screened. Thirteen were excluded owing to inadequate image quality, yielding a final study sample of 1487 patients (745 females, 742 males; mean age 25.21 ± 4.15 years) with 1491 impacted mandibular third molars. Four patients contributed bilateral impacted third molars; the remaining 1483 contributed a single impacted tooth. Eight teeth showing atypical Winter angulation were subsequently excluded from the logistic regression models owing to the absence of caries events in this subgroup, which precluded model convergence, leaving 1483 teeth in the regression analyses (Figure 1).

The distribution of Pell–Gregory classification was as follows: space classification, Class I 1027 (68.9%), Class II 306 (20.5%), and Class III 158 (10.6%); depth classification, Class A 856 (57.4%), Class B 447 (30.0%), and Class C 188 (12.6%). The distribution of Winter angulation is presented together with the corresponding caries and follicle-enlargement rates in Table 1.

### 3.2. Distal Caries of the Mandibular Second Molar

Regarding distal caries, neither the Pell–Gregory classification nor any other independent variable demonstrated a statistically significant association in univariate or multiple models (all *p* > 0.05; Table 2). Descriptively, caries was more frequent in female patients and in those with Class I and Class A impactions. Mesioangular impaction showed the highest descriptive caries rate (26.8%; Table 1), although this did not reach statistical significance. Class B depth impaction showed borderline trends in both the univariate model (OR 1.546, *p* = 0.057) and the multiple model (OR 1.688, *p* = 0.052), which may represent a clinically relevant pattern despite not achieving conventional significance thresholds.

**Table 2:** Binary logistic regression results for distal caries of the mandibular second molar (Pell–Gregory classification).

Variable	Caries Absent n (%)	Caries Present n (%)	Total n (%)	Univariate OR (95% CI)	*p*	Multiple OR (95% CI)	*p*
Age, mean ± SD (years)	25.19 ± 4.21	25.3 ± 3.91	25.21 ± 4.15	1.006 (0.975–1.038)	0.700	1.011 (0.978–1.044)	0.522
Female	603 (80.9)	142 (19.1)	745 (50.1)	1.013 (0.781–1.312)	0.925	1.029 (0.789–1.343)	0.831
Male (ref.)	602 (81.1)	140 (18.9)	742 (49.9)	Reference	—	Reference	—
Left side	586 (79.5)	151 (20.5)	737 (49.5)	1.220 (0.94–1.581)	0.134	1.202 (0.922–1.566)	0.173
Right side (ref.)	620 (79.5)	131 (17.4)	751 (50.5)	Reference	—	Reference	—
Pell–Gregory Class I	833 (81.1)	194 (18.9)	1027 (68.9)	1.020 (0.662–1.572)	0.929	0.890 (0.519–1.529)	0.674
Pell–Gregory Class II	247 (80.7)	59 (19.3)	306 (20.5)	1.046 (0.639–1.713)	0.858	0.798 (0.456–1.396)	0.428
Pell–Gregory Class III (ref.)	127 (81.4)	29 (18.6)	158 (10.6)	Reference	—	Reference	—
Pell–Gregory Class A	705 (82.4)	151 (17.6)	856 (57.4)	1.128 (0.735–1.731)	0.581	1.181 (0.687–2.030)	0.542
Pell–Gregory Class B	344 (77.3)	101 (22.7)	447 (30.0)	1.546 (0.987–2.423)	0.057	1.688 (0.995–2.864)	0.052
Pell–Gregory Class C (ref.)	158 (84.0)	30 (16.0)	188 (12.6)	Reference	—	Reference	—

Abbreviations: CI, confidence interval; OR, odds ratio; ref., reference category; SD, standard deviation. Note: Percentages for the Pell–Gregory space classification (Class I/II/III) have been recalculated against the total tooth-level sample (n = 1491) and now sum consistently with Table 3 (previously 100.1% owing to a rounding inconsistency).

### 3.3. Pericoronal Follicle Enlargement

Follicle enlargement was significantly associated with deeper Pell–Gregory impaction. In the adjusted model, Class I carried approximately 70% lower odds than Class III (OR 0.296; 95% CI 0.184–0.478; *p* < 0.001), and Class A carried approximately 75% lower odds than Class C (OR 0.254; 95% CI 0.161–0.400; *p* < 0.001). Younger age was an independent predictor (OR 0.964/year; 95% CI 0.933–0.996; *p* = 0.028). Winter angulation was not independently associated with follicle enlargement in the adjusted model (all *p* > 0.05; Table 3).

**Table 3:** Binary logistic regression results for pericoronal follicle enlargement (Winter and Pell–Gregory classifications).

Variable	Absent n (%)	Present n (%)	Total n (%) *	Univariate OR (95% CI)	*p*	Multiple OR (95% CI)	*p*
Age, mean ± SD (years)	25.66 ± 4.31	24.36 ± 3.71	25.21 ± 4.15	0.923 (0.897–0.949)	<0.001	0.964 (0.933–0.996)	0.028
Female	487 (65.0)	262 (35.0)	749 (50.2)	1.001 (0.810–1.239)	0.989	0.786 (0.609–1.013)	0.063
Male (ref.) ^†^	484 (65.1)	260 (34.9)	744 (49.8)	Reference	—	Reference	—
Left side	472 (63.9)	267 (36.1)	739 (49.6)	1.096 (0.886–1.356)	0.398	1.154 (0.897–1.485)	0.264
Right side (ref.)	496 (66.0)	256 (34.0)	752 (50.4)	Reference	—	Reference	—
Vertical	758 (74.3)	262 (25.7)	1020 (68.4)	0.207 (0.049–0.874)	0.032	0.418 (0.064–2.752)	0.364
Mesioangular	136 (52.1)	125 (47.9)	261 (17.5)	0.551 (0.129–2.355)	0.422	0.439 (0.067–2.880)	0.391
Horizontal	39 (37.5)	65 (62.5)	104 (7.0)	1.000 (0.226–4.417)	1.000	0.479 (0.071–3.228)	0.450
Distoangular	32 (32.7)	66 (67.3)	98 (6.6)	1.237 (0.278–5.504)	0.780	1.225 (0.177–8.465)	0.837
Atypical (ref.)	3 (37.5)	5 (62.5)	8 (0.5)	Reference	—	Reference	—
Pell–Gregory Class I	799 (77.8)	228 (22.2)	1027 (68.9)	0.140 (0.097–0.201)	<0.001	0.296 (0.184–0.478)	<0.001
Pell–Gregory Class II	117 (38.2)	189 (61.8)	306 (20.5)	0.792 (0.529–1.187)	0.259	0.889 (0.551–1.433)	0.628
Pell–Gregory Class III (ref.)	52 (32.9)	106 (67.1)	158 (10.6)	Reference	—	Reference	—
Pell–Gregory Class A	714 (83.8)	142 (16.6)	856 (57.4)	0.108 (0.076–0.153)	<0.001	0.254 (0.161–0.400)	<0.001
Pell–Gregory Class B	188 (42.1)	259 (57.9)	447 (30.0)	0.745 (0.523–1.061)	0.103	1.198 (0.771–1.861)	0.421
Pell–Gregory Class C (ref.)	66 (35.1)	122 (64.9)	188 (12.6)	Reference	—	Reference	—

Abbreviations: CI, confidence interval; OR, odds ratio; ref., reference category; SD, standard deviation. * Tooth-level analysis; totals reflect impacted teeth per sex subgroup, not patient counts. ^†^ Male total n = 744 (484 absent + 260 present); the sex subgroup denominator differs from the patient count (742 males) because 4 patients contributed bilateral teeth, both counted in sex subgroups.

### 3.4. Distribution of Winter Angulation

Table 1 presents the distribution of Winter angulation together with the corresponding distal-caries and follicle-enlargement rates. Vertical impaction was most prevalent (1020; 68.4%), followed by mesioangular (261; 17.5%), horizontal (104; 7.0%), distoangular (98; 6.6%), and atypical (8; 0.5%). No odds ratios for distal caries are presented for the Winter classification in Table 1 because logistic regression could not be computed owing to the absence of caries events in the atypical angulation group (n = 8), which precluded model convergence; only descriptive frequencies are therefore reported for this comparison, in contrast to the significance-tested results in Table 2 and Table 3.

## 4. Discussion

### 4.1. Principal Findings

This retrospective, STROBE-reported panoramic radiographic study evaluated 1487 patients with impacted mandibular third molars and examined the associations of both Winter angulation and Pell–Gregory classification with distal second molar caries and pericoronal follicle enlargement using multivariable logistic regression. To our knowledge, this is among the larger single-center retrospective studies to evaluate both classification systems and both outcomes simultaneously.

Vertical impaction predominated at 68.4% in our cohort, differing from global meta-analytic data reporting mesioangular impaction as the dominant pattern (41.17%) [2]. This discrepancy likely reflects population-specific characteristics, as vertical predominance has been consistently reported in Turkish population studies [23,24]. Mesioangular impaction showed the highest descriptive caries rate (26.8%), consistent with the published literature [14,15]. This did not reach statistical significance in our models, possibly reflecting the relatively modest subgroup size and the young mean cohort age (25.21 years).

Pell–Gregory Class I was the most prevalent space classification (68.9%), consistent with recent retrospective data [14,25]. Class A predominated among depth classifications (57.4%), consistent with contemporary studies [25,26].

### 4.2. Distal Caries

A central finding was the absence of a statistically significant association between impaction classification and distal caries. This contrasts with published meta-analytic data reporting an overall distal caries prevalence of approximately 29.89% [13], and with multicenter data showing that detection rates vary by third molar management strategy [16]. This apparent discrepancy warrants careful interpretation. Panoramic radiography has well-documented limitations in sensitivity for detecting proximal caries compared with bitewing radiography or CBCT [15,20]. It is therefore possible that the absence of a significant association reflects diagnostic under-detection rather than a true null effect, particularly given the relatively young cohort age (25.21 years) in which early-stage proximal caries may fall below the panoramic detection threshold. Additionally, important confounders including oral hygiene status, caries risk, periodontal condition, and smoking were not available in this retrospective dataset. The borderline association observed for Class B depth impaction (OR 1.688, *p* = 0.052) may represent a clinically relevant trend and warrants further investigation with more sensitive imaging modalities, such as CBCT or bitewing radiography, in a prospective design [21,22].

### 4.3. Pericoronal Follicle Enlargement

Pericoronal follicle enlargement was significantly associated with deeper Pell–Gregory impaction positions and younger age. These findings carry direct clinical and surgical relevance. Radiographically enlarged follicular spaces exceeding 2.5 mm may represent early cystic change, with histopathological studies confirming dentigerous cyst changes in up to 15.9% of radiographically normal follicles [17]. As discussed in Section 2.3, that histological validation was performed on CBCT rather than panoramic images; the consistency of our panoramic-based findings with this CBCT-anchored threshold, and with a separate panoramic study of 257 dentigerous cysts in which associated third molars were most frequently in Class III (64.6%) and horizontal positions [19], is reassuring but does not substitute for direct cross-modality validation, which we identified as a priority for future work. The association with younger age may reflect higher biological activity of the reduced enamel epithelium in younger individuals [17]. Importantly, enlarging follicular lesions in the mandibular angle region may also predispose to pathological fracture, particularly in the post-extraction period [6], further reinforcing the clinical importance of early identification and timely surgical management. Winter angulation was not independently associated with follicle enlargement in the adjusted model, suggesting that impaction depth and space are more informative predictors of follicular pathology than angulation alone.

### 4.4. Limitations

This study has several limitations. The retrospective design and exclusive reliance on panoramic radiography limit caries-detection sensitivity and three-dimensional follicular assessment; no distortion or magnification calibration and no direct CBCT cross-validation of the 2.5 mm follicular threshold were performed (Section 2.3). Formal intra-observer and inter-observer reliability statistics (e.g., ICC or Cohen’s kappa) could not be calculated, as individual evaluator scores were not separately recorded prior to consensus, and the individual contributions of the three examiners (two oral and maxillofacial surgeons and one doctoral candidate in oral and maxillofacial surgery) could therefore not be compared to characterize systematic differences between assessors. Analyses were conducted at the tooth level without formal adjustment for intra-patient clustering, although the impact was minimal given that only 4 out of 1487 patients (0.27%) contributed bilateral teeth; this bilateral-impaction rate is lower than typically reported in adult hospital populations and may reflect the single-tooth inclusion criterion or local referral patterns, as discussed in Section 2.4. Important confounders, including oral hygiene status, caries risk, periodontal condition, and smoking, were unavailable. The study population was relatively young and hospital-based, limiting generalizability. The atypical Winter group (n = 8) was too small for the logistic regression analysis of caries. Model diagnostics, including goodness-of-fit testing, were not available from the original SPSS output.

Future prospective studies should incorporate CBCT imaging and bitewing radiography alongside panoramic radiography to directly validate two-dimensional follicular and caries thresholds against three-dimensional and higher-sensitivity references; employ standardized, blinded intra- and inter-observer reliability protocols (ICC/Cohen’s kappa) with independently recorded pre-consensus scores; capture oral hygiene, periodontal, and caries-risk covariates; and apply multilevel modeling to formally account for intra-patient clustering in cohorts with a higher bilateral-impaction rate. Machine-learning-assisted radiographic classification is another promising avenue that could reduce inter-observer variability in impaction and follicular-space assessment.

## 5. Conclusions

In this retrospective, STROBE-reported radiographic study of 1491 impacted mandibular third molars, neither Winter angulation nor Pell–Gregory classification was significantly associated with distal second molar caries on panoramic radiography. However, deeper Pell–Gregory impaction positions (Class III/C) and younger age were independently associated with a significantly greater risk of pericoronal follicle enlargement. These findings support systematic radiographic classification and early clinical surveillance in young patients with deep impactions to guide individualized surgical decision-making. Future prospective studies incorporating CBCT imaging, bitewing radiography, clinical examination, and standardized inter-observer reliability protocols are recommended.

## Figures and Tables

**Table 1:** Distribution of Winter angulation and associated distal caries and follicle enlargement rates.

Winter Angulation	Total n (%)	Caries Absent n (%)	Caries Present n (%)	Follicle Absent n (%)	Follicle Present n (%)	OR Caries ^‡^
Vertical	1020 (68.4)	847 (83.0)	173 (17.0)	758 (74.3)	262 (25.7)	N/A
Mesioangular	261 (17.5)	191 (73.2)	70 (26.8)	136 (52.1)	125 (47.9)	N/A
Horizontal	104 (7.0)	78 (75.0)	26 (25.0)	39 (37.5)	65 (62.5)	N/A
Distoangular	98 (6.6)	82 (83.7)	16 (16.3)	32 (32.7)	66 (67.3)	N/A
Atypical	8 (0.5)	8 (100.0)	0 (0.0)	3 (37.5)	5 (62.5)	N/A
Total	1491 (100)	1206 (80.9)	285 (19.1)	968 (64.9) ^§^	522 (35.0) ^§^	—

^‡^ Logistic regression for Winter classification in relation to distal caries could not be computed owing to the absence of caries events in the atypical angulation group (n = 8, 0 caries events), which precluded model convergence; descriptive rates are provided for all angulation groups, and this is why, unlike Table 2 and Table 3, no *p*-values are presented here. ^§^ Follicle data available for 1490 out of 1491 teeth; 1 tooth had missing follicle assessment data.

## Data Availability

The data presented in this study are available on request from the corresponding author owing to institutional data-sharing restrictions on patient radiographic records.

## Supplementary Materials

The following supporting information can be downloaded at: https://www.mdpi.com/article/10.3390/tomography12090122/s1, Table S1: STROBE Statement Checklist.
