# Supplementary material for: The Association Between Mandibular Third Molar Impaction, Distal Caries of Second Molars, and Pericoronal Follicle Enlargement: A Retrospective Panoramic Radiographic Study
Source: Tomography. 2026 Aug 25;12(9):122. doi: 10.3390/tomography12090122 (PMC13611545; doi:10.3390/tomography12090122)
Supplement: Supplementary file 1 [file tomography-12-00122-s001.zip › tomography-4444308-supplementary.pdf]

Table S1. STROBE Statement — Checklist of items for cross-sectional/retrospective observational studies

Manuscript: The Association Between Mandibular Third Molar Impaction, Distal Caries of Second Molars, and Pericoronal Follicle Enlargement: A Retrospective Panoramic Radiographic Study (tomography-4444308)

Item	No.	Location	Recommendation / how addressed
Title and abstract	1	Title; Abstract	(a) Indicated as a retrospective (cross-sectional) study in the title. (b) Abstract provides an informative summary of design, methods, and results.
Introduction — Background/rationale	2	Sec. 1, ¶1–5	Scientific background and rationale for the investigation.
Introduction — Objectives	3	Sec. 1, last ¶	Specific objectives and hypotheses stated.
Methods — Study design	4	Sec. 2.1	Retrospective radiographic (cross-sectional) design stated.
Methods — Setting	5	Sec. 2.2	Setting, location, and period of recruitment described (Public Hospitals Services Directorate ADSM/ADH Region 2).
Methods — Participants	6	Sec. 2.2, 3.1	Eligibility criteria and sources/methods of selection of participants; numerical outcomes of screening in Sec. 3.1 and Figure 2.
Methods — Variables	7	Sec. 2.3	Outcomes (distal caries, follicle enlargement), exposures (Winter, Pell–Gregory), and confounders (age, sex, side) defined.
Methods — Data sources/measurement	8	Sec. 2.3	Sources of data and measurement methods described for each variable; limitations of the 2.5 mm threshold explicitly discussed.
Methods — Bias	9	Sec. 2.3, 2.4, 4.4	Potential sources of bias described: lack of inter-observer reliability testing, low bilateral-impaction rate, panoramic-only imaging.
Methods — Study size	10	Sec. 3.1	Explanation of study size (1,500 screened; final n reported).
Methods — Quantitative variables	11	Sec. 2.3	Handling of quantitative variables described (age as continuous; classification systems as categorical).
Methods — Statistical methods	12	Sec. 2.5	(a) Statistical methods described, including regression. (b) Subgroup handling described. (c) Missing data: 1 tooth with missing follicle data noted (Table 3 footnote). (d)/(e) Not applicable (no matching/sampling strategy beyond stated inclusion criteria).
Results — Participants	13	Sec. 3.1, Figure 2	Numbers of individuals at each stage reported in flow diagram.
Results — Descriptive data	14	Sec. 3.1, 3.4, Tables 1–3	Characteristics of participants and exposures described.
Results — Outcome data	15	Sec. 3.2, 3.3	Numbers of outcome events (caries, follicle enlargement) reported.
Results — Main results	16	Sec. 3.2, 3.3, Tables 1–2	(a) Unadjusted and adjusted estimates with 95% CI reported. (b) Not applicable. (c) Not applicable.
Results — Other analyses	17	Sec. 3.4, Table 3	Descriptive Winter-classification analyses reported; explained why no regression was possible for the atypical subgroup.

Discussion — Key results	18	Sec. 4.1	Key results summarized with reference to study objectives.
Discussion — Limitations	19	Sec. 4.4	Limitations discussed in detail, including direction and magnitude of potential bias.
Discussion — Interpretation	20	Sec. 4.2, 4.3	Cautious overall interpretation considering objectives, limitations, and multiplicity of analyses.
Discussion — Generalizability	21	Sec. 4.4	Generalizability discussed (young, hospital-based population).
Other information — Funding	22	Funding statement	Source of funding and role of funders described (none).
